# Protective Value of Aspirin Loading Dose on Left Ventricular Remodeling After ST-Elevation Myocardial Infarction

**DOI:** 10.3389/fcvm.2022.786509

**Published:** 2022-03-16

**Authors:** Camilla Calvieri, Nicola Galea, Francesco Cilia, Giacomo Pambianchi, Giuseppe Mancuso, Domenico Filomena, Sara Cimino, Iacopo Carbone, Marco Francone, Luciano Agati, Carlo Catalano

**Affiliations:** ^1^Department of Clinical, Internal, Anesthesiological and Cardiovascular Sciences, “Policlinico Umberto I” Hospital, Sapienza University of Rome, Rome, Italy; ^2^Department of Experimental Medicine, Sapienza University of Rome, Rome, Italy; ^3^Department of Radiological, Oncological and Pathological Sciences, Sapienza University of Rome, Rome, Italy; ^4^Department of Biomedical Sciences, Humanitas University, Milan, Italy; ^5^Humanitas Research Hospital, Istituti di Ricovero e Cura a Carattere Scientifico (IRCCS), Milan, Italy

**Keywords:** myocardial infarction, feature tracking, cardiac magnetic resonance, myocardial strain, aspirin, ventricular remodeling

## Abstract

**Aims:**

Left ventricular (LV) remodeling after ST-elevation myocardial infarction (STEMI) is a complex process, defined as changes of LV volumes over time. CMR feature tracking analysis (CMR-FT) offers an accurate quantitative assessment of LV wall deformation and myocardial contractile function. This study aimed to evaluate the role of myocardial strain parameters in predicting LV remodeling and to investigate the effect of Aspirin (ASA) dose before primary coronary angioplasty (pPCI) on myocardial injury and early LV remodeling.

**Methods and Results:**

Seventy-eight patients undergoing CMR, within 9 days from symptom onset and after 6 months, were enrolled in this cohort retrospective study. We divided the study population into three groups based on a revised Bullock's classification and we evaluated the role of baseline CMR features in predicting early LV remodeling. Regarding CMR strain analysis, worse global circumferential and longitudinal strain (GCS and GLS) values were associated with adverse LV remodeling. Patients were also divided based on pre-pPCI ASA dosage. Significant differences were detected in patients receiving ASA 500 mg dose before pPCI, which showed lower infarct size extent and better strain values compared to those treated with ASA 250 mg. The stepwise multivariate logistic regression analysis, adjusted for covariates, indicated that a 500 mg ASA dose remained an inverse independent predictor of early adverse LV remodeling.

**Conclusion:**

GCS and GLS have high specificity to detect early LV adverse remodeling. We first reported a protective effect of ASA loading dose of 500 mg before pPCI on LV myocardial damage and in reducing early LV adverse remodeling.

## Introduction

Left ventricular (LV) remodeling after ST-elevation myocardial infarction (STEMI) is a complex and multifactorial process, beginning within the first week and lasting almost until 1 year (1). Despite improvements in dual antiplatelet therapy and the reperfusion strategies of STEMI patients, adverse LV remodeling remains an open issue predisposing patients to poor cardiovascular outcomes (2, 3). “Early” LV adverse remodeling, an LV ventricular maladaptive process occurring until 6 months after myocardial infarction, has been widely described (4, 5), whereas few studies have also described reverse remodeling in those patients (6). The latter is a favorable LV remodeling process, which has been correlated with good clinical outcomes (2, 6). Cardiac magnetic resonance (CMR) is widely considered the gold-standard imaging modality to assess LV volumes and LV ejection fraction (LVEF), to quantify myocardial infarct size (IS), and to predict improvement in regional and global contractile function (2, 4). Different thresholds have been proposed to classify LV remodeling by using CMR (3, 7). Recently, Bulluck et al. have proved that a cut off of 12% of volume increase in left ventricular end-diastolic volume (LVEDV) and/or left ventricular end-systolic volume (LVESV) is more accurate to detect early LV remodeling, compared to other cut-offs (7). This criterion has been proved to identify a high-risk group of STEMI patients for the composite end point of all-cause mortality and heart failure (HF) at a median follow-up of 5.8 years (8). Moreover, different factors assessed by CMR within the first week of an acute STEMI, such as LVEF, IS, and microvascular obstruction (MVO), have been reported to be independent strong predictors of LV adverse remodeling and clinical outcomes after STEMI (9, 10). The quantitative assessment of myocardial contractile deformation enabled by CMR feature tracking analysis (CMR-FT) offers deeper information on regional and global LV function other than LVEF, helping to predict LV remodeling (11, 12). Furthermore, CMR-FT has been increasingly used in STEMI patients to assess the efficacy of cardioprotective therapies in preventing post-infarction LV remodeling (12–14), such as to evaluate the effect of intravenous administration of metoprolol on long term prognostic effect in the randomized METOCARD-CNIC trial (13).

Among cardioprotective drugs, antiplatelet agents play a pivotal role in improving myocardial reperfusion after STEMI (15). To date, a wide dose range of aspirin (ASA) is recommended by current guidelines before primary percutaneous coronary intervention (pPCI) in STEMI patients (16). However, it has been poorly investigated whether the pre-pPCI ASA dose may have an impact on LV remodeling.

The present study aimed to confirm the predictive role of myocardial strain parameters by CMR-FT in detecting LV adverse remodeling and assess the effect of ASA dose before pPCI on IS extent, LV global contractility, and adverse LV remodeling in a population of reperfused STEMI.

## Methods

### Study Design

This is a retrospective single-center observational study. All STEMI patients included in this study were treated by pPCI within 12 h after symptoms onset and had undergone CMR in the early post-infarction phase (within 9 days from hospital admission) and after 6 months (FU). We retrospectively selected participants from our Institution's STEMI CMR registry of 177 patients, 78 patients, admitted to our Coronary Care Unit between January 2010 and February 2019, who met the inclusion criteria (10).

Demographic, clinical, and pharmacological data were registered in the emergency room before any drug administration and before pPCI reperfusion. STEMI was defined as previously reported (16). Body mass index (BMI) was measured in each patient and we defined obesity as BMI > 25 kg/m^2^. Cardiac troponin I levels were measured within 12 h of admission with acute myocardial infarction defined according to ESC guidelines (16). The normal range for troponin I was between 0 and 0.04 ng/mL. Among angiographic parameters: culprit lesion infarct related artery, TIMI flow before and after PCI, time to reperfusion, pre- and post-pPCI systolic blood pressure (SBP), and diastolic blood pressure (DBP) were collected. Post-pPCI SBP, DBP, and HR values were measured within 2 h post-PCI. The above parameters were not evaluated at the CMR time (neither in the first exam nor at follow-up). Exclusion criteria were previously reported (10).

For LV early remodeling categories, we divided the population into three groups, based on a revised Bullock's classification scheme for LV remodeling (7) by comparing baseline and 6-month follow-up CMR (FU-CMR) exams: “*adverse remodeling*” defined as an increase of LVEDV and/or LVESV > 12%, “*reverse remodeling*” as a decrease of 12% of LVESV, and “*null remodeling*” as other volume percentage increase or decrease < 12%.

To evaluate the effect of ASA loading dose on IS and LV remodeling, we analyzed a subgroup of 45 STEMI patients, according to oral ASA dose at hospital admission before PCI (we included only patients who received a traceable ASA dose). We divided the population into two groups, one receiving 250 mg and the other treated with a loading dose of 500 mg. ASA dosage was decided according to the preference of the emergency department physicians who treated the patients.

This study complied with the Declaration of Helsinki. All participants gave written informed consent to the protocol and the study was approved by the local ethical committee.

Patients or the public were not involved in the design, conduct, reporting, or dissemination plans of our research.

### CMR Imaging Protocol and Analysis

CMR exams were performed by using a 1.5-T scanner (Magnetom Avanto, Siemens Healthcare) equipped with a multichannel phase-array cardiac coil. A standardized CMR protocol, including the ECG-gated cine steady-state free precession (cine-MR), T2-weighted short tau inversion recovery (T2w-STIR) for the evaluation of area-at-risk (AAR), and late gadolinium enhancement sequences (LGE) for infarct size (IS), was performed in all patients. Technical parameters and image analysis details have been previously described (10, 17). Image analysis was performed using dedicated commercial software (cvi42 v. 5.3, Circle Cardiovascular Imaging, Calgary, AB, Canada). LV remodeling index (LV-RI) was obtained by dividing LV mass for LVEDV at baseline and FU-CMR exam. Myocardial salvage was quantified by the difference between AAR and IS (18, 19). Feature tracking (CMR-FT) analysis was performed offline using dedicated commercial software (cvi42 v. 5.3, Circle Cardiovascular Imaging, Calgary, AB, Canada) as detailed elsewhere (11). CMR-FT analysis was applied using basal, mid, and apical ventricular short-axis, vertical-, and horizontal long-axis views in cine-SSFP images. LV endocardial and epicardial contours were traced in a semiautomatic way on short- and long-axis cine-SSFP images in all the phases of the cardiac cycle. LV myocardial tracking was then visually reviewed and the contouring errors were corrected and the analysis repeated. We measured global radial (GRS), circumferential (GCS), and longitudinal (GLS) components of the different LV strain parameters to detect any alteration in myocardial fibers deformability during the cardiac cycle. Furthermore, all measurements of CMR parameters (left and right ventricular volumes, AAR, IS, MVO, GRS, GCS, and GLS) were performed in consensus between two observers with 15 (N.G.) and 6 years (F.C.) of experience in CMR. Intra-observer and inter-observer variability for measurements of GRS, GCS, and GLS were assessed in a sample of 10 patients; two investigators measured blinded the same exam, and one investigator repeated the analysis 1 week later, blinded to the previous measurements. To assess the inter- and intra-observer variability Wilcoxon signed rank test and the intraclass correlation coefficients (ICC) were determined for each parameter (GCS, GLS, GRS).

### Statistical Analysis

All continuous variables were assessed for normality with the Shapiro-Wilk test and by examination of their histogram. Data are presented as frequencies and percentages, mean ± standard deviation (SD) or median and interquartile ranges (IQRs), as appropriate. Comparison of continuous variables was performed using the one-way ANOVA test with the *post-hoc* Bonferroni test or Kruskal-Wallis test, when appropriate. Unadjusted differences between two continuous variables were compared using the Wilcoxon Sum Rank Test or *t*-test di Student, as appropriate. Comparison of normally distributed data between baseline and FU-CMR was performed using paired *t*-test. Correlation between parameters was assessed using either Pearson's correlation coefficient (r) or Spearman's rank coefficient (ρ), where appropriate. Differences in categorical variables were compared using the Chi Square test or Fisher exact test, as appropriate. Comparisons of the CMR-derived parameters (deformation parameters, ventricular volumes, and LVEF) between different groups were evaluated by analysis of covariance (ANCOVA) with Bonferroni-adjusted *post-hoc* tests, after adjusting for age, sex, body mass index (BMI). Interclass correlation coefficients were calculated to assess inter-observer and intra-observer agreement of LV volumes and strain measurements. The primary end-point was defined as LV adverse remodeling. Receiver operating characteristic (ROC) analysis was performed to compare AAR, MVO, LGE, and myocardial strain values in detecting LV adverse remodeling and assess their predictive power. Continuous variables were dichotomized based on Youden's J statistic to allow for comparison of the odds ratios (ORs) in the univariate and multivariate models. In order to identify predictors of LV adverse remodeling, univariate and multivariate logistic regression models were used. Variables with a *p* < 0.10 at univariate logistic analysis were subsequently introduced into the multivariate logistic regression model. To select the best fit, the stepwise logistic regression method was applied. Associations between the investigated variables and the likelihood of LV adverse remodeling were estimated using hazard ratios (OR) and their 95% confidence intervals (95% CI). The Hosmer-Lemeshow test was performed to show the good calibration of the logistic regression model. Only *p* < 0.05 were considered statistically significant. All tests were 2-tailed and analyses were performed using computer software packages (SPSS-24.0, IBM, NY, USA).

## Results

**Figure 1:**
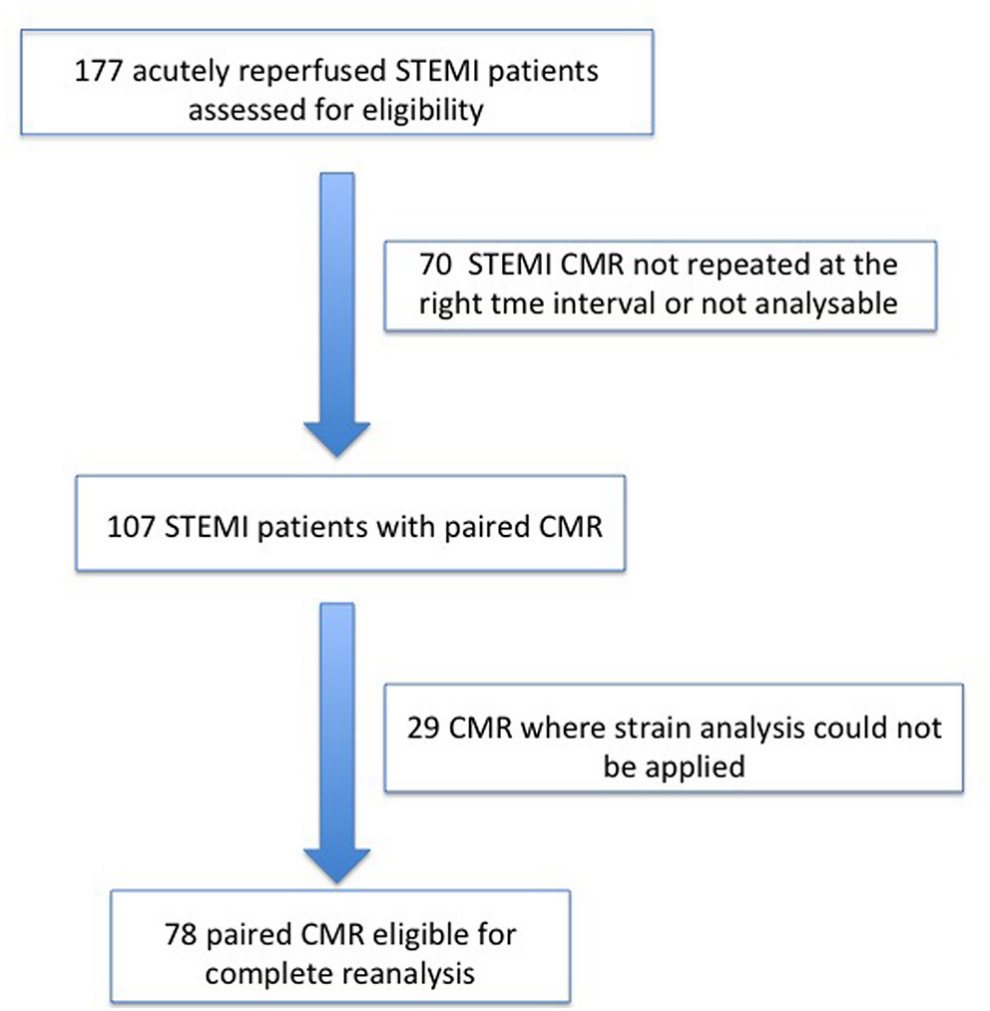
Recruitment flow chart. STEMI, ST-elevation myocardial infarction; CMR, Cardiac magnetic resonance.

**Figure 2:**
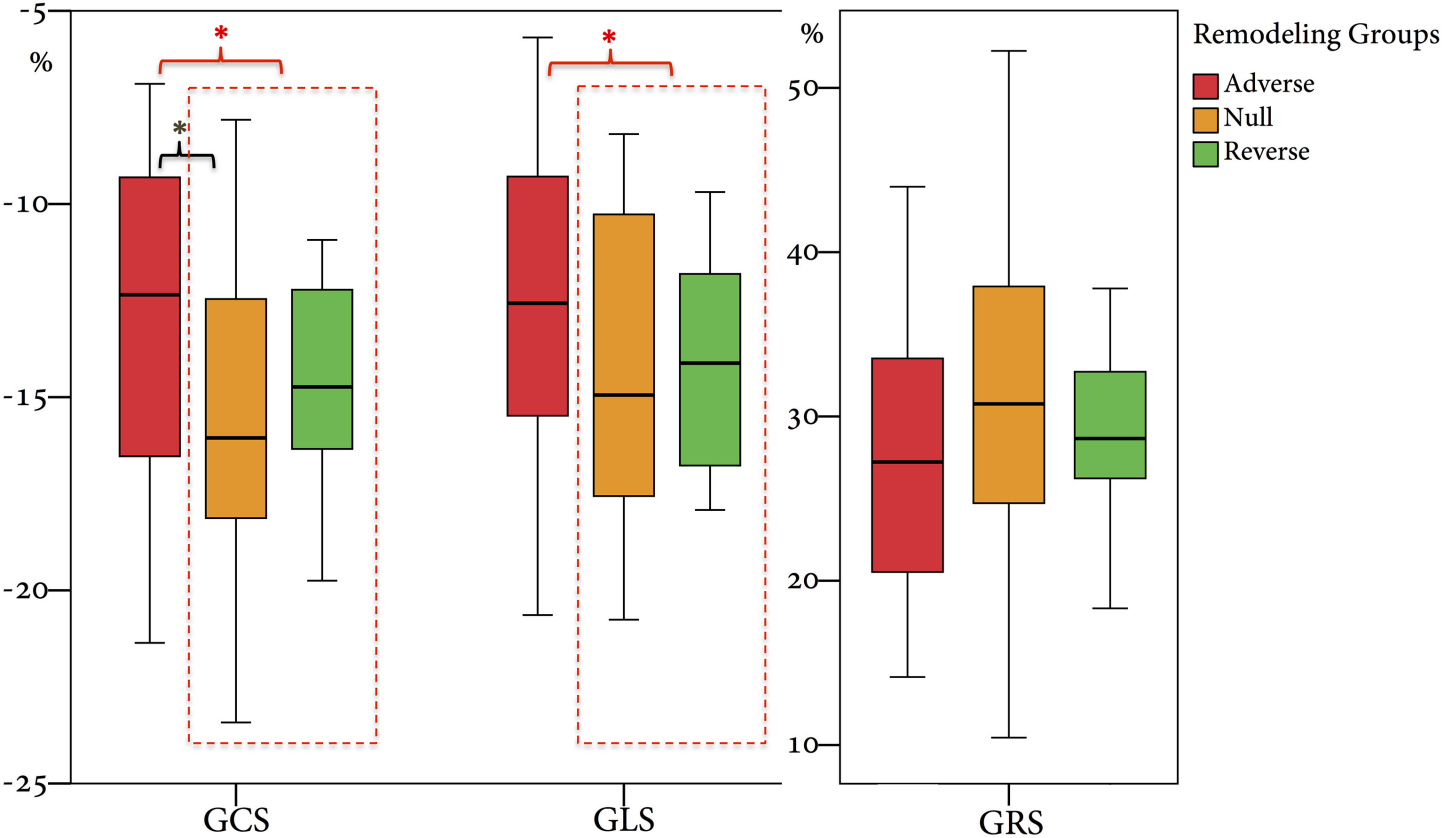
Comparison of GRS, GCS, and GLS values according to the three remodeling groups. GRS, global radial strain; GCS, global circumferential strain; GLS, global longitudinal strain. ^*^ symbol indicates statistically significant comparisons among groups. ^*^p < 0.05.

From 2010 to 2019, a total of 1,285 STEMI patients underwent pPCI in Policlinico Umberto I Hospital, of which ~15% had CMR performed and 78 were recruited as reported in the flow chart (Figure 1). According to our internal procedures, when allowed by the clinical condition of the patient, the availability of the scanner/staff, and in the absence of contraindications, CMR was performed in reperfused STEMI patients for the assessment of post-infarction myocardial injury during the acute phase and to detect complications at short and long term follow up. Among the study population of enrolled STEMI patients, 38 (49%) showed adverse, 22 (28%) reverse, and 18 (23%) null LV remodeling, assessed by paired CMR exams (baseline and FU-CMR). Demographic, clinical, angiographic, and pharmacological characteristics of the STEMI population according to the three groups are illustrated in Table 1. No differences in demographic data, cardiovascular risk factors, angiographic presentation, or laboratory findings were found. Agreement in strain measurements was excellent with ICC between 0.922 and 0.943 (*p* < 0.001, Supplementary Material) and *p* > 0.43 (range 0.43–0.85) at Wilcoxon's test. Regarding pharmacological therapy, no differences were noted about beta-blocker, antidiabetics, statins, ACE-inhibitors, spironolactone, GbIIb-IIIa inhibitors, and clopidogrel use among the three LV remodeling categories, except for the pre-pPCI ASA administration. At the first CMR exam, patients with LV adverse remodeling showed higher AAR, IS, and MVO, if compared to the other two groups (Table 2). Meanwhile, at FU-CMR, the adverse remodeling group had lower LVEF, greater IS extent, and lower LV-RI. At CMR strain analysis, significantly worse GCS and GLS values at baseline and FU-CMRs in the adverse LV remodeling group, compared to the sum of the other two groups, were found (Figure 2, Table 2). After adjusting for age, sex, BMI by ANCOVA analysis, the only CMR derived parameters that reported a different statistical significance if compared with ANOVA analysis, according to the three remodeling groups, were baseline AAR, baseline MVO, FU IS, baseline GRS, and baseline GLS.

**Table 1:** Demographic, clinical, pharmacological, and angiographic characteristics according to LV remodeling groups.

	**Adverse** **remodeling** **(*n* = 38)**	**Reverse** **remodeling** **(*n* = 22)**	**Null** **remodeling** **(*n* = 18)**	***p*-value**
**Demographic data and CAD comorbidities**				
Male sex *n*, %	36 (95)	21 (95)	16 (89)	0.646
Age yrs	59 ± 13	54 ± 10	58 ± 12	0.289
BSA m^2^	1.88 ± 0.2	1.97 ± 0.2	1.95 ± 0.1	0.215
BMI kg/m^2^	25.6 ± 3.7	27.9 ± 4.5	26.05 ± 3	0.089
Hypertension *n*, %	15 (40)	12 (55)	11 (61)	0.304
Obesity *n*, %	24 (63.2)	15 (68.2)	12 (66.7)	0.917
Dislipidemia *n*, %	20 (54)	7 (32)	11 (61)	0.133
CAD familiarity *n*, %	23 (62)	14 (64)	13 (72)	0.755
Diabetes *n*, %	5 (13)	2 (9)	2 (11)	0.874
Smoking habitus *n*, %	21 (58)	14 (64)	10 (56)	0.881
**pPCI characteristics**				
LAD IRA *n*, %	23 (60)	11 (50)	13 (72)	0.568
LCX IRA *n*, %	7 (18)	5 (23)	1 (6)	
RCA IRA *n*, %	8 (21)	6 (27)	4 (22)	
Thromboaspiration *n*, %	20 (71)	8 (53)	6 (46)	0.241
Time to reperfusion min	95 (55–165)	120 (90–256)	120 (95–266)	0.197
TIMI flow pre-pPCI 0 *n*, %	21 (72)	15 (88)	9 (64)	0.259
TIMI flow pre-pPCI 1 *n*, %	8 (28)	2 (12)	4 (29)	
TIMI flow post-pPCI 2 *n*, %	1 (3)	0 (0)	0 (0)	0.583
TIMI flow post-pPCI 3 *n*, %	31 (97)	18 (100)	16 (100)	
SBP pre-pPCI mmHg	125 ± 24	132 ± 21	141 ± 24	0.071
DBP pre-pPCI mmHg	77 ± 13	80 ± 12	85 ± 11	0.183
HR pre-pPCI bpm	70 (62–80)	60 (65–77)	78 (65–90)	0.517
SBP post-pPCI mmHg	130 ± 15	126 ± 18	131 ± 16	0.674
DBP post-pPCI mmHg	79 ± 9	78 ± 12	76 ± 13	0.856
HR post-pPCI bpm	75 (65–85)	72 (70–80)	75 (59–80)	0.600
**Pharmacological therapy before and after pPCI**				
ASA 250 mg *n*, %	15 (71.4)	5 (45.5)	3 (23.1)	**0.021**
ASA 500 mg *n*, %	6 (28.6)	6 (54.5)	10 (77)	
Clopidogrel *n*, %	25 (66)	16 (73)	14 (78)	0.632
GbIIbIIa inhibitors *n*, %	15 (54)	10 (63)	7 (54)	0.833
ACE-inibitors *n*, %	31 (86)	21 (96)	17 (94)	0.406
Spironolactone *n*, %	19 (53)	11 (50)	9 (50)	0.971
β-blockers *n*, %	32 (91)	20 (91)	18 (100)	0.428
Statins *n*, %	33 (92)	22 (100)	18 (100)	0.176
Antidiabetics *n*,%	3 (7.9)	1 (4.5)	1 (5.6)	0.989
**Laboratory parameters**				
Hb g/dl	13.7 ± 1.8	14.2 ± 1.3	14 ± 1.8	0.635
CK MB peak ng/mL	163.2 ± 127	150.4 ± 141	192.2 ± 228	0.707
TnI peak ng/ml	30.3 ± 44	9.3 ± 17	37.5 ± 80	0.165
Creatinine mg/dL	0.88 ± 0.2	0.92 ± 0.2	0.89 ± 0.2	0.784

*CAD, coronary artery disease; BSA, body mass index; pPCI, primary percutaneous coronary intervention; LAD, left anterior descending artery; IRA, infarct related artery; LCX, left circumflex artery; RCA, right coronary artery; TIMI, Thrombolysis in Myocardial Infarction; SBP, systolic blood pressure; DBP, diastolic blood pressure; HR, heart rate; bpm, beat per minute; ASA, Aspirin; ACE, angiotensin converting enzyme; Hb, hemoglobin; CK MB, creatine kinase isoenzymes MB; TnI, troponin I. Bold values are those statistically significant, as well as those with a p value < 0.05*.

**Table 2:** CMR parameters at baseline and 6-month follow-up, according to LV remodeling categories.

	**Adverse remodeling** **(*n* = 38)**	**Reverse remodeling** **(*n* = 22)**	**Null remodeling** **(*n* = 18)**	***p*** ^**A vs. R**^	***p*** ^**R vs. N**^	***p*** ^**A vs. N**^	***p*** ^**A vs. (R + N)**^	***p*** ^**overall**^
LVEDVi at baseline, ml/m^2^	69.3 (55–76)	66.8 (55–82)	72.3 (61–79)	0.939	0.765	0.563	0.749	0.866
LVESVi at baseline, ml/m^2^	34 (26–50)	33 (28–42)	36 (29–39)	0.902	0.935	0.687	0.757	0.940
LVMi at baseline, gr/m^2^	60.7 (54–73)	61.5 (54–71)	58.8 (44–71)	0.899	0.414	0.207	0.415	0.470
LVEF at baseline, %	46 ± 12	48 ± 9	51 ± 7	1.000	0.863	0.191	0.126	0.177
LV-RI at baseline	0.96 ± 0.2	0.93 ± 0.2	0.86 ± 0.2	1.000	0.907	0.242	0.156	0.215
AAR at baseline, %	30.6 ± 19	19.3 ± 14	18.4 ± 13	0.052	1.000	0.065	**0.004**	**0.017**
IS at baseline, %	18.5 (12.5–29.6)	10.8 (4.2–19.7)	9.9 (6.2–21.9)	**0.004**	0.663	**0.027**	**0.002**	**0.007**
Myocardial salvage, %	9.3 ± 18	7.6 ± 10	6.02 ± 9	0.700	0.627	0.512	0.497	0.755
MVO at baseline, %	0.78 (0.08–3.31)	0.83 (0.075–1.44)	0.24 (0.0–0.79)	0.410	0.207	**0.017**	**0.046**	0.058
LVEDVi at FU, ml/m^2^	87.7 (75–105)	54.9 (48–69)	72.5(61–78)	**<0.001**	**0.011**	**0.005**	**<0.001**	**<0.001**
LVESVi at FU, ml/m^2^	42.4 (33–65)	24.3 (20–31)	34.9(30–41)	**<0.001**	**0.002**	**0.028**	**<0.001**	**<0.001**
LVMi at FU, gr/m^2^	59.4 (51–73)	55.6 (47–62)	56.1 (49–64)	0.221	0.688	0.262	0.149	0.347
LVEF at FU, %	46 ± 12	56 ± 8	50 ± 8	**0.005**	0.288	0.821	**0.007**	**0.006**
LV-RI at FU	0.69 ± 0.2	1.01 ± 0.2	0.82 ± 0.2	**<0.001**	**0.018**	0.084	**<0.001**	**<0.001**
IS at FU, gr	12.8 (8.5–24.4)	7.2 (3.5–15.4)	10 (5.8–19.5)	**0.007**	0.413	0.090	**0.006**	**0.016**
GRS at baseline, %	26.9 ± 8.2	29.4 ± 5	30.9 ± 9	0.699	0.475	0.198	0.068	0.154
GRS at FU,%	28.8 ± 9	31.4 ± 7	33.4 ± 8	0.762	0.418	0.184	0.074	0.153
GCS at baseline, %	−12.9 ± 4	−14.7 ± 2	−15.5 ± 4	0.225	0.426	**0.048**	**0.012**	**0.034**
GCS at FU, %	−14.2 ± 4	−16.4 ± 3	−16.8 ± 3	0.130	0.668	0.074	**0.010**	**0.036**
GLS at baseline, %	−12.4 ± 4	−14.0 ± 3	−14.8 ± 4	0.338	0.483	0.087	**0.023**	0.063
GLS at FU, %	−13.9 ± 4	−16.0 ± 3	−15.3 ± 4	0.141	0.494	0.676	**0.049**	0.122

*A, adverse remodeling; R, reverse remodeling; N, null remodeling; B, baseline; FU, follow-up; LVEDVi, left ventricular end-diastolic volume index; LVESVi, left ventricular end-systolic volume index; LVMi, Left ventricular mass index; LVEF, left ventricular ejection fraction; LV-RI, left ventricular remodeling index; AAR, area at risk; IS, infarct size; FU, follow-up; MVO, microvascular obstruction; LGE, late gadolinium enhancement; GRS, global radial strain; GCS, global circumferential strain; GLS, global longitudinal strain. Bold values are those statistically significant, as well as those with a p value < 0.05*.

**Figure 3:**
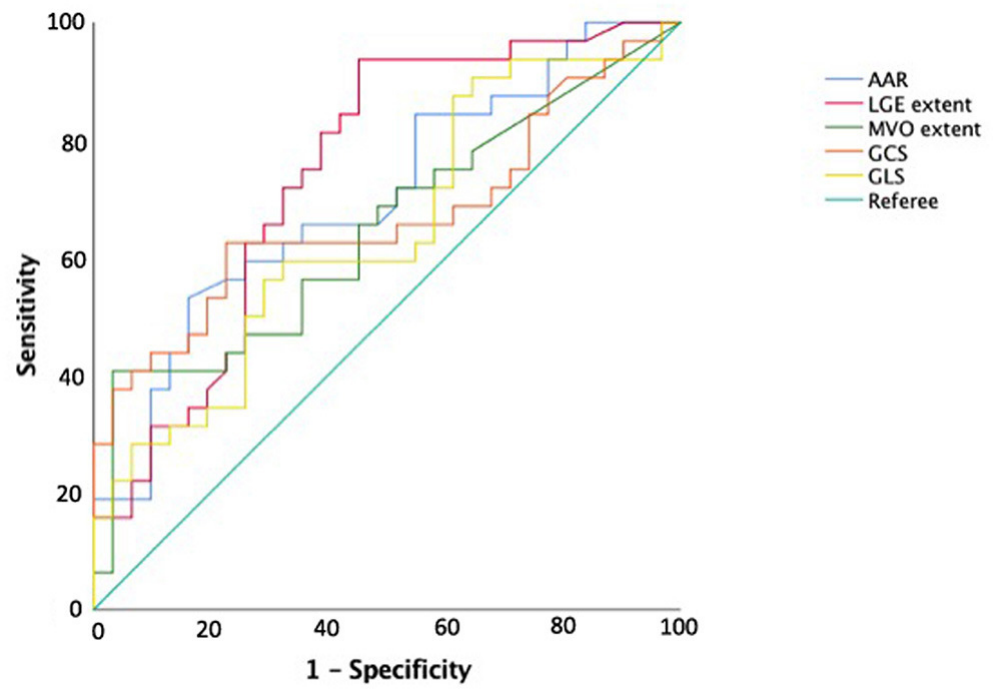
Receiver operating curve analysis for predicting adverse left ventricular remodeling. AAR, area-at-risk; LGE, late gadolinium enhancement; MVO, microvascular obstruction; GRS, global radial strain; GCS, global circumferential strain; GLS, global longitudinal strain.

At ROC curve analysis (Figure 3), AAR, IS, MVO, GLS, and GCS values, measured at baseline CMR, showed significant sensitivity (Se) and specificity (Sp) to detect adverse remodeling at FU-CMR (AAR AUC 0.684, 95% CI 0.563–0.789, *p* = 0.0040 Youden index J 0.3476 with Se: 51% and Sp: 83%; LGE AUC 0.747, 95% CI 0.635–0.859, *p* < 0.001 Youden index J for LGE > 9.7% with Se: 93% and Sp: 40%; MVO AUC 0.637, 95% CI 0.514–0.747, *p* = 0.0405 Youden index J for MVO 0.39 gr with a Se: 42% and Sp: 97%; GLS AUC 0.639, 95% CI 0.521–0.745, *p* = 0.0306 Youden index J > −10.21 % with Se: 35% and Sp: 90%; GCS AUC 0.660 95% CI 0.543–0.764, *p* = 0.0138 Youden index J > −11.09 % with Se: 43% and Sp: 92%). In pairwise comparisons, there were no significant differences between the AUC of ROC curves of the measured CMR parameters by DeLong et al. (20) method (*p* > 0.05 for all). About infarct related artery, significantly worse GCS and GLS were observed in those having, as the culprit lesion, the left anterior descending artery (LAD) compared to circumphlex (LCX) and right coronary arteries (RCA) (GCS values in LAD −13.0 ± 4 vs. −16.0 ± 3% in LCX vs. −15.1±3% in RCA, *p* = 0.014; GLS values −12 ± 4% in LAD vs. −16.1 ± 3% in LCX vs. −15.4 ± 2% in RCA, *p* < 0.001).

### ASA Loading Dose Effects

**Figure 4:**
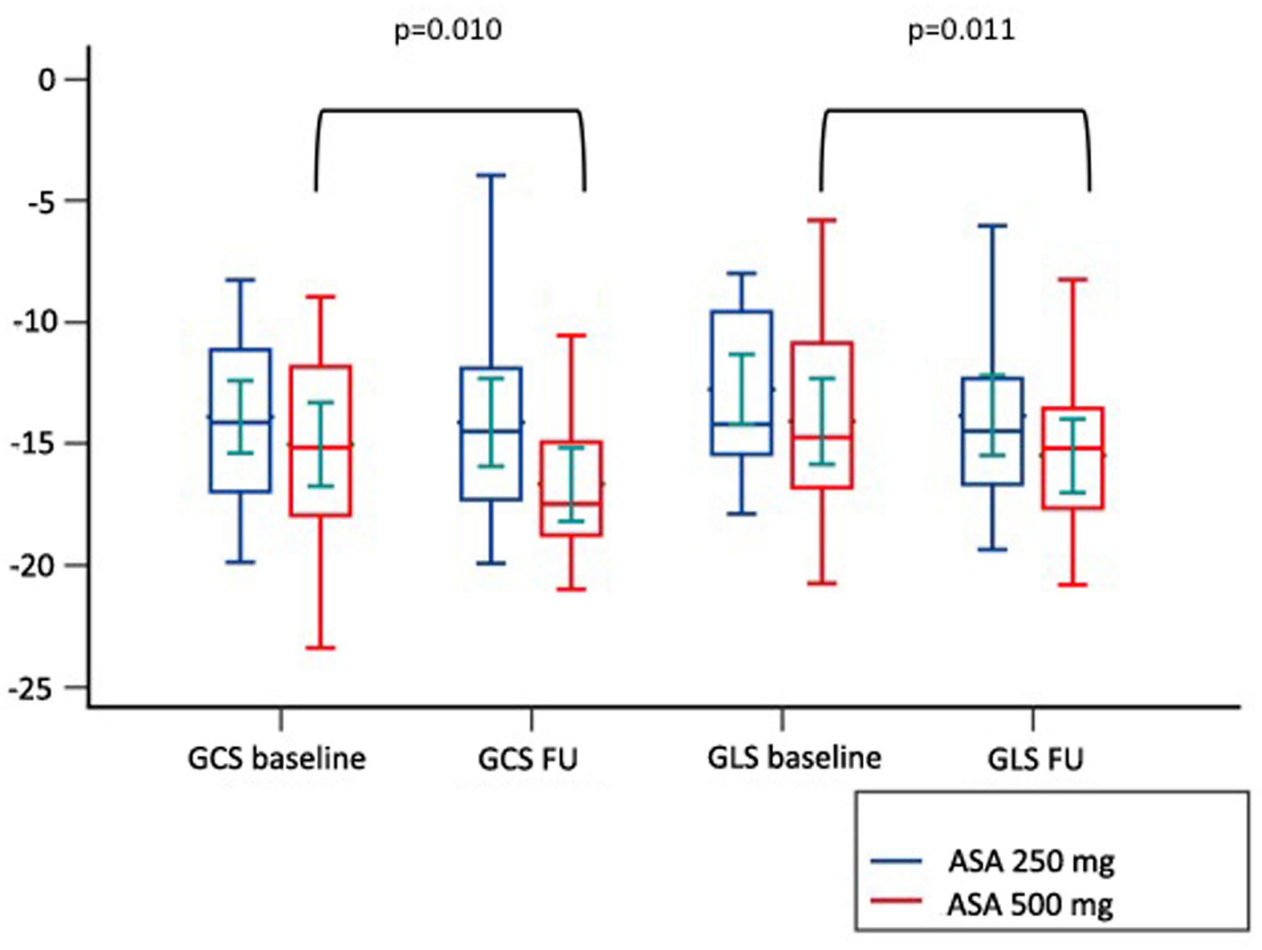
Comparison of paired GLS and GCS values between CMR at baseline and at follow up according to pre-pPCI ASA loading dose. GCS, global circumferential strain; GLS, global longitudinal strain; ASA, aspirin.

Among cardiovascular drugs administered before PCI, patients with LV adverse remodeling were less frequently treated with ASA loading dose (500 mg), compared to those with reverse or null remodeling (Table 1). Thus, we decided to assess the ASA loading dose effect according to LV remodeling groups. Among the 45 STEMI patients who received a traceable dose of ASA before PCI, 22 STEMI underwent to ASA loading dose, and 23 patients to ASA 250 mg. No differences according to demographic and clinical variables between the two groups were found (Table 3). Patients receiving ASA loading dose before PCI had lower MVO and lower IS extent at baseline and FU-CMR and better strain values (GRS and GCS) compared to those treated with ASA 250 mg (Table 4). Moreover, in the ASA loading group a significant improvement of GCS and GLS, but not GRS values, between baseline and FU-CMR exams (GCS −15.09 ± 3.8 to −16.7 ± 3.2%, *p* = 0.010, GLS −14.1 ± 3.9 to −15.7 ± 3.2%, *p* = 0.011, GRS 31.0 ± 7.8 to 32.9 ± 7%, *p* = 0.104) was noted. Meanwhile, no significant differences of GCS and GLS values between two paired CMR exams within the group treated with lower ASA doses (GCS −13.8 ± 3.5 to −14.1 ± 3.9% *p* = 0.612, GLS −12.9 ± 3.3 to −13.8 ± 3.7%, *p* = 0.232, GRS 28.2 ± 6.6 to 27.4 ± 7.9%, *p* = 0.594) were observed (Figure 4).

**Table 3:** Demographic, clinical, pharmacological, and angiographic characteristics according to the ASA group.

	**ASA 250 mg** **(*n* = 23)**	**ASA 500 mg** **(*n* = 22)**	***p*-value**
Male sex *n*, %	22 (95.7)	20 (90.9)	0.524
Age yrs	59 (51–65)	54 (46–68)	0.658
BSA m^2^	1.96 (1.79–2.01)	1.94(1.82–2.07)	0.708
BMI kg/m^2^	25.9 ± 3.2	26.6 ± 3.8	0.561
Hypertension *n*, %	13 (56.5)	11 (52.4)	0.783
Obesity *n*,%	17 (73.9)	13 (59.1)	0.292
Dislipidemia *n*, %	14 (60.9)	10 (47.6)	0.378
CAD familiarity *n*, %	17 (73.9)	11 (52.4)	0.138
Diabetes *n*, %	4 (17.4)	2 (10.0)	0.669
Smoking habitus *n*, %	17 (77.3)	14 (66.7)	0.438
LAD *n*, %	16 (69.5)	15 (68.2)	0.862
LCX *n*, %	4 (17.4)	3 (13.6)	
RCA *n*, %	3 (13)	4 (18.2)	
Thromboaspiration *n*, %	15 (65.2)	8 (36.4)	**0.047**
Time to reperfusion min	120 (90–210)	120 (80–163)	0.445
TIMI flow pre PCI 0 *n*, %	17 (73.9)	11 (50)	0.098
TIMI flow pre PCI 1 *n*, %	6 (26.1)	11 (50)	
TIMI flow post PCI 2 *n*, %	5 (21.7)	3 (13.6)	0.477
TIMI flow post PCI 3 *n*, %	18 (78.3)	19 (86.4)	
SBP pre PCI mmHg	130 (112–150)	135 (124–151)	0.510
DBP pre PCI mmHg	80 (70–89)	80 (77–96)	0.412
HR pre PCI bpm	74 (65–81)	67 (61–77)	0.153
SBP post PCI mmHg	130 (120–135)	132 (111–147)	0.522
DBP post PCI mmHg	75 (70–80)	75 (70–88)	0.800
HR post PCI bpm	75 (65–82)	73 (63–80)	0.463
Hb g/dl	13.7 (12.5–15)	13.9 (12.7–15.5)	0.426
CK MB peak ng/mL	123.6 (67–213)	107.3 (26–333)	0.876
TnI peak ng/ml	3.8 (1.9–27.2)	4.4 (0.7–10.7)	0.669

*CAD, coronary artery disease; BSA, body mass index; pPCI, primary percutaneous coronary intervention; LAD, left anterior descending artery; IRA, infarct related artery; LCX, left circumflex artery; RCA, right coronary artery; TIMI, Thrombolysis in Myocardial Infarction; SBP, systolic blood pressure; DBP, diastolic blood pressure; HR, heart rate; bpm, beat per minute; ASA, Aspirin; ACE, angiotensin converting enzyme; Hb, hemoglobin; CK MB, creatine kinase isoenzymes MB; TnI, troponin I. Bold values are those statistically significant, as well as those with a p value < 0.05*.

**Table 4:** CMR parameters at baseline and 6-month follow-up, according to ASA groups.

	**ASA 250 mg** **(*n* = 23)**	**ASA 500 mg** **(*n* = 22)**	***p*-value**
LVEDVi_B_ ml/m^2^	69.2 (54–80)	73.0 (60–82)	0.892
LVESVi_B_ ml/m^2^	36.4 (25–49)	35.8 (27–42)	0.716
LVMi_B_ gr/m^2^	54.7 (49–65)	59.5 (48–75)	0.716
LVEF_B_ %	46 ± 10	50 ± 9	0.284
LV-RI_B_	0.9 ± 0.2	0.9 ± 0.2	0.502
LVEF_FU_ %	47.5 ± 10	52.6 ± 10	0.100
LVMi_FU_ gr/m^2^	54.9 (46–69)	57.5 (48–64)	0.880
LV-RI_FU_	0.76 ± 0.2	0.82 ± 0.2	0.342
Adverse remodeling *n*, %	15 (65)	6 (27)	**0.011**
AAR_B_ %	28.4 ± 12	29.4 ± 12	0.888
MVO_B_ %	0.7 (0.09–2.26)	0.59 (0.00–1.57)	0.555
IS_B_ %	19.0 (12–29)	12.0 (6–19.7)	**0.031**
Myocardial salvage %	7.2 (1.9–13.4)	10.4 (2.8–17.4)	**0.375**
IS_FU_ %	12.6 (9–28)	7.3 (4–17)	**0.009**
GRS_B_ %	28.1 ± 6	31.1 ± 8	0.157
GRS_FU_ %	27.4 ± 8	32.9 ± 7	**0.022**
GCS_B_ %	−13.9 ± 3	−15.2 ± 4	0.240
GCS_FU_ %	−14.1 ± 4	−16.7 ± 3	**0.028**
GLS_B_ %	−12.8 ± 3	−14.2 ± 4	0.204
GLS_FU_ %	−13.8 ± 4	−15.7 ± 3	0.094

*ASA, Aspirin; A, adverse remodeling; R, reverse remodeling; N, null remodeling; B, baseline; FU, follow-up; LVEDVi, left ventricular end-diastolic volume index; LVESVi, left ventricular end-systolic volume index; LVMi, Left ventricular mass index; LVEF, left ventricular ejection fraction; LV-RI, left ventricular remodeling index; AAR, area at risk; IS, infarct size; FU, follow-up; MVO, microvascular obstruction; LGE, late gadolinium enhancement; GRS, global radial strain; GCS, global circumferential strain; GLS, global longitudinal strain. Bold values are those statistically significant, as well as those with a p value <0.05*.

The stepwise multivariate logistic regression analysis was adjusted for IS, GCS, and GLS cut-offs and related to adverse remodeling. An ASA loading dose of 500 mg remained an inverse independent predictor of early adverse LV remodeling (Table 5). The Hosmer-Lemeshow test demonstrated good calibration of the logistic regression model (χ^2^ = 1.095, *p* = 0.955).

**Table 5:** Stepwise multivariate logistic regression analysis of predictors of LV adverse remodeling.

	**Univariate analysis**				**Multivariate analysis**			
	**O.R**.	**Lower**	**upper**	***p*-value**	**O.R**	**Lower**	**upper**	***p*-value**
ASA 500 mg	0.243	0.073	0.811	0.021	0.243	0.073	0.811	0.021
IS	10.254	2.702	38.912	0.001				
GCS	6.857	2.023	23.243	0.002				
GLS	3.069	1.022	9.216	0.046				
Thromboasp	2.500	0.828	7.548	0.104				
Time to reperfusion	0.998	0.995	1.001	0.207				

*ASA, aspirin; IS, infarct size; GCS, global circumferential strain at baseline CMR; GLS, global longitudinal strain at baseline CMR; Thromboasp, thromboaspiration*.

## Discussion

Our study confirmed the value of the strain analysis provided by CMR-FT in assessing the risk of LV early adverse remodeling, according to Bulluck's definition (7). In particular, we demonstrated that cut-offs of −11.09% for GCS and −10.21 for GLS, measured at baseline CMR, are highly specific in predicting LV adverse remodeling whereas those parameters do not differ between patients with null and reverse remodeling. Although both strain CMR parameters have been used previously to predict adverse LV remodeling (11, 12, 21), they have never been applied to these new categories (7). Furthermore, we retrospectively evaluated the effect of ASA dose before pPCI in STEMI patients for the first time, and described a positive effect of a loading dose of 500 mg on early LV remodeling.

### CMR Parameters in Predicting LV Remodeling

**Figure 5:**
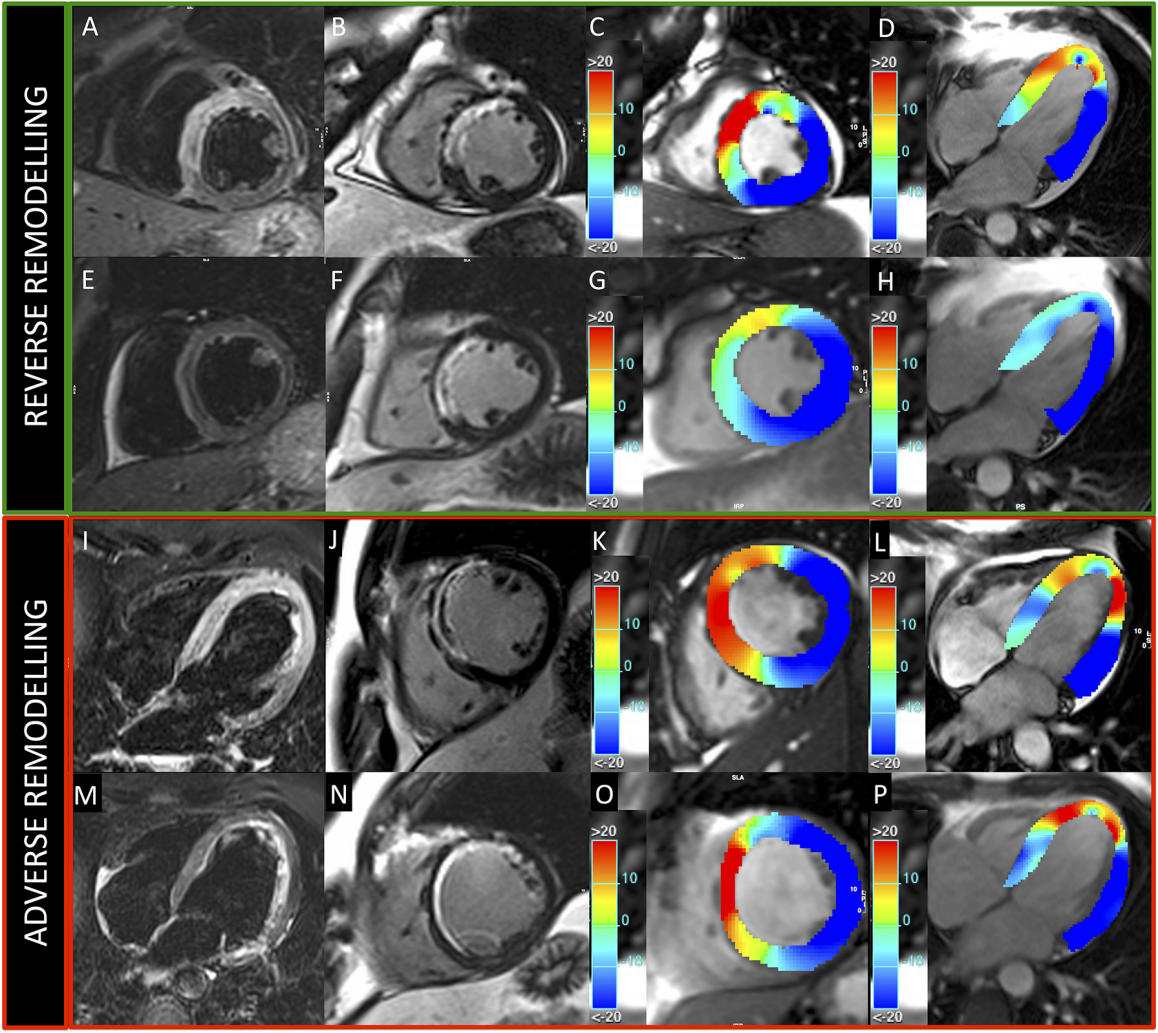
Patients presenting reverse and adverse remodeling at baseline and follow-up CMRs. Images acquired in two patients with reverse (upper green panel) and adverse (lower red panel) remodeling, treated, respectively, with 500 and 250 mg ASA dose. Upper and lower rows represent exams acquired at baseline and 6-month follow-up, respectively. T2-weighted **(A,E,I,M)** and LGE **(B,F,J,N)** images show anterior myocardial infarction in both cases. GCS **(C,G,K,O)** and GLS **(D,H,L,P)** colored maps demonstrate better baseline values and higher recovery of myocardial contractility at follow-up in the patient with reverse remodeling.

In this study, we categorized the population using the three LV remodeling patterns based on Bulluck's definition (7), observing a similar percentage distribution (adverse remodeling 49 vs. 45%, reverse remodeling 28 vs. 29%, and null remodeling 19 vs. 23%). For CMR parameters, the adverse remodeling group showed greater AAR, IS, and MVO at baseline CMR and greater IS at FU-CMR, as already reported (7). Otherwise, no differences in IS and MVO between reverse and null remodeling groups were found in our study. Notably, there were no differences in salvage myocardium extent among the three groups. Although IS and MVO are known predictors of adverse remodeling (7), the relationship between them and myocardial recovery is still an open issue (6, 7). Moreover, at FU-CMR, the adverse remodeling group showed lower LVEF, LV-RI, and greater IS, as compared to the other two groups. Regarding FT-CMR strain analysis, the adverse remodeling group showed worse GCS and GLS values at baseline and FU-CMR (Figure 5), if compared to reverse and null remodeling categories as one. Various studies investigated the value of FT-CMR features in predicting adverse remodeling (11, 12, 22), using different cut-off values and follow-up periods. For the definition of adverse LV remodeling, we considered LVEDV and/or an LVESV delta change of 12%, which is lower than most reports, and 6 months for follow-up, longer if compared to other studies, mostly around 3/4 months. Thus, this issue may have induced a larger rate of adverse remodeling in our population (49%), as compared to other cohorts (17–24.4%) (11, 12, 22, 23). In the majority of studies, baseline GLS was the best predictor of adverse remodeling among all strain values (11, 22–24). In particular, Reindl et al. (22) reported significant differences in baseline strain values and infarct size/MVO percentage between no remodeling and remodeling groups, as observed in our cohort. Moreover, they showed that a GLS-value > −14% was the best independent predictor of 4 months LV adverse remodeling (LVEDV delta change of 10%) with an AUC of 0.610, which do not differ substantially from our GLS AUC value of 0.639 using LVEDV/LVESV delta change of 12%. In the retrospective study of Cha et al. (11), at ROC curve analysis (AUC: 0.756, 95% CI = 0.636–0.887, *p* < 0.001), the GLS cut-off of −12.84% resulted in high sensitivity (Se: 85%) and low specificity (Sp: 61%) in predicting adverse remodeling at 6 months (LVEDV delta change of 20%), whereas in our study the optimal cut-off was lower (GLS > −10.21%), with lower sensitivity (Se: 35%) and higher specificity (Sp: 90%), likely reflecting the different criteria in classifying the remodeling groups. Interestingly, in our study GCS was the strongest predictor of adverse remodeling among the baseline strain values, as already reported by other authors (12, 25, 26). Holmes et al. (12) found that GCS was a superior predictor of LV adverse remodeling at 3 months follow-up than MVO, GLS, and GRS, although they considered a cohort of both STEMI and non-STEMI patients (12), differently from our population of STEMI only. Similarly, Buss et al. demonstrated that GCS is useful in predicting preserved LV function at 6 months follow-up but they did not evaluate LV remodeling groups (25).

For the other CMR factors, we compared strain parameters and IS in predicting adverse remodeling, demonstrating high sensitivity for IS cut-off of 9.7% (Se: 93%), whereas the GCS and GLS may offer high specificity at the optimal cut-off values (Sp: 92 and 90%, respectively). These results support the evidence that both GCS and GLS values are both useful in the prognostic stratification of STEMI patients and should be interpreted in combination with other CMR parameters, to improve risk profiling and tailoring of therapies.

Lastly, we observed worse GCS and GLS values in patients with LAD culprit artery, compared to LCX and RCA, even if no difference of infarct related artery distribution according to LV remodeling groups was noted. These findings confirmed that the anterior location of myocardial infarction may be responsible for a greater myocardial injury and function impairment, as reported by Masci et al. (27).

### Protective Effect of 500 mg Aspirin Administration Prior to PCI

Regarding clinical and procedural characteristics, among the LV remodeling groups, a strong difference in pre-pPCI ASA loading dose was found, suggesting its potentially protective role in preventing early adverse remodeling. Besides, a lower IS extent in patients who received a 500 mg dose of ASA at baseline and FU-CMR was found. These findings are in agreement with those reported in elective procedures, where an ASA reload of 325 mg before PCI improved reperfusion indices, reduced myocardial “no reflow” and periprocedural myocardial injury, by blunting post-PCI increase of thromboxane B_2_ production (28). Moreover, a higher ASA dose before revascularization was also associated with an improvement of LVEF (28). A possible explanation could be due to the effect of high ASA doses in inhibiting post-ischemic LV remodeling process (29), by suppression of proinflammatory cytokines, reduction of collagen deposition, and left ventricular hypertrophy as demonstrated in animal models (30). Indeed, Muller et al. (31) observed a reduction of collagen production and LV hypertrophy in a model of angiotensin II induced organ damage in rats pretreated with 600 mg of aspirin, reporting that high ASA doses decreased mortality, cardiac hypertrophy, fibrosis, and albuminuria independently of blood pressure, by inhibition of IKK/NF-κB pathway. Meanwhile, Adamek et al. (29) demonstrated that high ASA doses, after left coronary artery ligation, suppressed inflammatory response in animal experiments, by inhibiting proinflammatory cytokines such IL-1β and TNF, even though not affecting LV remodeling.

As previously reported, a longer time to reperfusion as well as poor reperfusion indices can determine myocardial injury and LV adverse remodeling (19). In our study, although the greater percentage of thrombo-aspiration might explain the higher rate of adverse remodeling in the lower ASA dose group, an ASA loading dose of 500 mg remained, at multivariate analysis, a significant protective factor independently from IS.

Furthermore, for the first time, ASA's role in improving myocardial contractility at FU-CMR has been examined. As FT-CMR has been demonstrated to be a valid and accurate tool to assess short and long term efficacy of cardioprotective drugs such as metoprolol (13, 32), no data about the effect of ASA on early LV remodeling and myocardial strain have been reported previously. Indeed, few clinical studies investigated the optimal ASA dose before pPCI in STEMI patients (16), which remains to be defined. In a retrospective study by Berger et al., the higher ASA dose in STEMI patients was associated with a greater risk of moderate-to-severe bleeding than lower doses, but there was no difference in 30 day mortality or ischemic related outcomes (33). The CURRENT-OASIS 7 trial compared open-label high-dose (300–325 mg daily) to low-dose (75–100 mg) aspirin in patients with acute coronary syndromes undergoing planned PCI (34). In a recent retrospective study by Rossi et al. (35), a high intravenous ASA dose in STEMI patients increased in-hospital mortality if compared to patients treated with intravenous low doses or oral doses, but it did not influence cardiovascular events after 1-year follow up. Recently, a clinical randomized trial demonstrated that an intravenous ASA dose of 250–500 mg compared to 300 mg orally can lead to complete inhibition of thromboxane generation and platelet aggregation, indicating the efficacy of higher doses in ameliorating myocardial reperfusion (36). The effect of higher ASA doses in ameliorating IS and contractility and in preventing LV adverse remodeling in our study may be interpreted by a complete inhibition of platelet cicloxigenase 1 activity (COX1) (28). Indeed, as demonstrated in the REMODELING prospective cohort trial in STEMI patients treated with antiplatelet agents, increased levels of platelet activation and inflammation may be responsible for post-STEMI LV adverse remodeling processes (37). High platelet reactivity is frequent after STEMI and is associated with major adverse cardiovascular and cerebral events, especially if associated with P2Y12 inhibitors platelet activity (38). Moreover, plasma thromboxane B_2_ has been reported to have a role in modulating myocardial reperfusion and has been hypothesized to have a vasoconstriction effect, modulated by COX-1 (39).

In our study, the protective effect of ASA high loading doses in preventing adverse LV remodeling was independent of IS and myocardial strain values at multivariate analysis. Nonetheless, if confirmed, these data could have a relevant clinical impact, with potential implications on the clinical course and prognosis of a large number of STEMI patients. Therefore, further pharmacological interventional clinical trials that are adequately powered and possibly multicenter in approach, with a larger population and longer follow-up, are required.

## Limitations

This study has several limitations. First, it is a retrospective and not an interventional-randomized study about ASA dose before pPCI. ASA dose before pPCI has been arbitrarily decided by the emergency room physician or cardiologist on call, introducing a selection bias. Second, considering the nature of the study, no preliminary statistical power analysis was performed and in particular, the statistical significance of the prognostic value of ASA loading dose at multivariate analysis should be interpreted with caution. Third, due to the vast heterogeneity of the study population, we cannot exclude the interference of the effect of other pre-existing drug treatments (in particular chronic administration of ASA). Third, no laboratory assessment of platelet aggregation was performed because it was not foreseen in our routine clinical practices; therefore, it could be only hypothesized that the positive effects of ASA loading dose are mediated by the complete inhibition of Cox1. Fourth, in our study, we considered only global strain values, which represent validated and reproducible indices of the whole LV contractile function. Regional strain analysis was not included in the analysis, therefore it was not possible to assess whether the positive effect of ASA is exerted directly on the infarcted tissue, in the peri-infarct region, or the remote tissue.

## Conclusion

Our findings reported that CMR strain parameters (GCS and GLS) have high specificity to detect early adverse remodeling, compared to other CMR imaging indices (IS, AAR, and MVO). The present study described a protective effect of 500 mg ASA loading dose before pPCI in improving LV contractility, myocardial damage, and in reducing early LV adverse remodeling for the first time. Further randomized multicentric studies using CMR analysis are needed to clarify this issue.

## Data Availability Statement

The raw data supporting the conclusions of this article will be made available by the authors, without undue reservation.

## Ethics Statement

The studies involving human participants were reviewed and approved by Policlinico Umberto I Hospital - Rome. The patients/participants provided their written informed consent to participate in this study.

## Author Contributions

CCal, NG, MF, and LA conceived and designed the study. CCal, FC, DF, and SC collected the clinical, laboratory, and CMR data. NG, GP, GM, FC, and IC performed and independently analyzed CMR exams. NG, FC, and MF interpreted CMR results. CCal performed statistical analyses. CCal, NG, MF, LA, and CCat interpreted the results. CCal, NG, and FC co-wrote the manuscript and prepared the figures. IC, MF, LA, and CCat contributed to the revision of the manuscript. All authors have read and approved the final manuscript.

## Funding

Publication fee was funded by Sapienza University of Rome – Progetti di Ricerca Prot. RM11916B88D7811D.

## Conflict of Interest

The authors declare that the research was conducted in the absence of any commercial or financial relationships that could be construed as a potential conflict of interest.

## Publisher's Note

All claims expressed in this article are solely those of the authors and do not necessarily represent those of their affiliated organizations, or those of the publisher, the editors and the reviewers. Any product that may be evaluated in this article, or claim that may be made by its manufacturer, is not guaranteed or endorsed by the publisher.

## References

[1] Cohn JN Ferrari R Sharpe N. Cardiac remodeling–concepts and clinical implications: a consensus paper from an international forum on cardiac remodeling. Behalf of an International Forum on cardiac remodeling. J Am Coll Cardiol. (2000) 35:569–82. 10.1016/S0735-1097(99)00630-0 10716457

[2] Funaro S La Torre G Madonna M Galiuto L Scara A Labbadia A. Incidence, determinants, and prognostic value of reverse left ventricular remodelling after primary percutaneous coronary intervention: results of the Acute Myocardial Infarction Contrast Imaging (AMICI) multicenter study. Eur Heart J. (2009) 30:566–75. 10.1093/eurheartj/ehn529 19098019 PMC2649283

[3] Rodriguez-Palomares JF Gavara J Ferreira-Gonzalez I Valente F Rios C Rodriguez-Garcia J. Prognostic value of initial left ventricular remodeling in patients with reperfused STEMI. JACC Cardiovasc Imaging. (2019) 12:2445–56. 10.1016/j.jcmg.2019.02.025 31202752

[4] Masci PG Pavon AG Pontone G Symons R Lorenzoni V Francone M. Early or deferred cardiovascular magnetic resonance after ST-segment-elevation myocardial infarction for effective risk stratification. Eur Heart J Cardiovasc Imaging. (2020) 21:632–9. 10.1093/ehjci/jez179 31326993

[5] Canali E Masci P Bogaert J Bucciarelli Ducci C Francone M McAlindon E. Impact of gender differences on myocardial salvage and post-ischaemic left ventricular remodelling after primary coronary angioplasty: new insights from cardiovascular magnetic resonance. Eur Heart J Cardiovasc Imaging. (2012) 13:948–53. 10.1093/ehjci/jes087 22531464

[6] Bodi V Monmeneu JV Ortiz-Perez JT Lopez-Lereu MP Bonanad C Husser O. Prediction of reverse remodeling at cardiac MR imaging soon after first st-segment-elevation myocardial infarction: results of a large prospective registry. Radiology. (2016) 278:54–63. 10.1148/radiol.2015142674 26348232

[7] Bulluck H Go YY Crimi G Ludman AJ Rosmini S Abdel-Gadir A. Defining left ventricular remodeling following acute ST-segment elevation myocardial infarction using cardiovascular magnetic resonance. J Cardiovasc Magn Reson. (2017) 19:26. 10.1186/s12968-017-0343-9 28285594 PMC5346848

[8] Bulluck H Carberry J Carrick D McEntegart M Petrie MC Eteiba H. Redefining adverse and reverse left ventricular remodeling by cardiovascular magnetic resonance following st-segment-elevation myocardial infarction and their implications on long-term prognosis. Circ Cardiovasc Imaging. (2020) 13:e009937. 10.1161/CIRCIMAGING.119.009937 32689822

[9] Carrick D Haig C Rauhalammi S Ahmed N Mordi I McEntegart M. Prognostic significance of infarct core pathology revealed by quantitative non-contrast in comparison with contrast cardiac magnetic resonance imaging in reperfused ST-elevation myocardial infarction survivors. Eur Heart J. (2016) 37:1044–59. 10.1093/eurheartj/ehv372 26261290 PMC4816961

[10] Galea N Dacquino GM Ammendola RM Coco S Agati L De Luca L. Microvascular obstruction extent predicts major adverse cardiovascular events in patients with acute myocardial infarction and preserved ejection fraction. Eur Radiol. (2019) 29:2369–77. 10.1007/s00330-018-5895-z 30552479

[11] Cha MJ Lee JH Jung HN Kim Y Choe YH Kim SM. Cardiac magnetic resonance-tissue tracking for the early prediction of adverse left ventricular remodeling after ST-segment elevation myocardial infarction. Int J Cardiovasc Imaging. (2019) 35:2095–102. 10.1007/s10554-019-01659-w 31267265

[12] Holmes AA Romero J Levsky JM Haramati LB Phuong N Rezai-Gharai L. Circumferential strain acquired by CMR early after acute myocardial infarction adds incremental predictive value to late gadolinium enhancement imaging to predict late myocardial remodeling and subsequent risk of sudden cardiac death. J Interv Card Electrophysiol. (2017) 50:211–8. 10.1007/s10840-017-0296-9 29143170

[13] Podlesnikar T Pizarro G Fernandez-Jimenez R Montero-Cabezas JM Sanchez-Gonzalez J Bucciarelli-Ducci C. Effect of early metoprolol during ST-segment elevation myocardial infarction on left ventricular strain: feature-tracking cardiovascular magnetic resonance substudy from the METOCARD-CNIC trial. JACC Cardiovasc Imaging. (2019) 12:1188–98. 10.1016/j.jcmg.2018.07.019 30219400

[14] Bulluck H Hammond-Haley M Weinmann S Martinez-Macias R Hausenloy DJ. Myocardial infarct size by CMR in clinical cardioprotection studies: insights from randomized controlled trials. JACC Cardiovasc Imaging. (2017) 10:230–40. 10.1016/j.jcmg.2017.01.008 28279370 PMC5348096

[15] Sardella G Sangiorgi GM Mancone M Colantonio R Donahue M Politi L. A multicenter randomized study to evaluate intracoronary abciximab with the ClearWay catheter to improve outcomes with Lysis (IC ClearLy): trial study design and rationale. J Cardiovasc Med. (2010) 11:529–35. 10.2459/JCM.0b013e3283341c1c 19918189

[16] Ibanez B James S Agewall S Antunes MJ Bucciarelli-Ducci C Bueno H. 2017 ESC Guidelines for the management of acute myocardial infarction in patients presenting with ST-segment elevation: the Task Force for the management of acute myocardial infarction in patients presenting with ST-segment elevation of the European Society of Cardiology (ESC). Eur Heart J. (2018) 39:119–77. 10.1093/eurheartj/ehx393 28886621

[17] Symons R Masci PG Francone M Claus P Barison A Carbone I. Impact of active smoking on myocardial infarction severity in reperfused ST-segment elevation myocardial infarction patients: the smoker's paradox revisited. Eur Heart J. (2016) 37:2756–64. 10.1093/eurheartj/ehv738 26804461

[18] Francone M Carbone I Agati L Bucciarelli Ducci C Mangia M Iacucci I. Utility of T2-weighted short-tau inversion recovery (STIR) sequences in cardiac MRI: an overview of clinical applications in ischaemic and non-ischaemic heart disease. Radiol Med. (2011) 116:32–46. 10.1007/s11547-010-0594-0 20927650

[19] Francone M Bucciarelli-Ducci C Carbone I Canali E Scardala R Calabrese FA. Impact of primary coronary angioplasty delay on myocardial salvage, infarct size, and microvascular damage in patients with ST-segment elevation myocardial infarction: insight from cardiovascular magnetic resonance. J Am Coll Cardiol. (2009) 54:2145–53. 10.1016/j.jacc.2009.08.024 19942086

[20] DeLong ER DeLong DM Clarke-Pearson DL. Comparing the areas under two or more correlated receiver operating characteristic curves: a nonparametric approach. Biometrics. (1988) 44:837–45. 3203132

[21] Khan JN Singh A Nazir SA Kanagala P Gershlick AH McCann GP. Comparison of cardiovascular magnetic resonance feature tracking and tagging for the assessment of left ventricular systolic strain in acute myocardial infarction. Eur J Radiol. (2015) 84:840–8. 10.1016/j.ejrad.2015.02.002 25743248

[22] Reindl M Tiller C Holzknecht M Lechner I Eisner D Riepl L. Global longitudinal strain by feature tracking for optimized prediction of adverse remodeling after ST-elevation myocardial infarction. Clin Res Cardiol. (2021) 110:61–71. 10.1007/s00392-020-01649-2 32296969

[23] Shetye AM Nazir SA Razvi NA Price N Khan JN Lai FY. Comparison of global myocardial strain assessed by cardiovascular magnetic resonance tagging and feature tracking to infarct size at predicting remodelling following STEMI. BMC Cardiovasc Disord. (2017) 17:7. 10.1186/s12872-016-0461-6 28056808 PMC5217595

[24] Garg P Kidambi A Swoboda PP Foley JR Musa TA Ripley DP. The role of left ventricular deformation in the assessment of microvascular obstruction and intramyocardial haemorrhage. Int J Cardiovasc Imaging. (2017) 33:361–70. 10.1007/s10554-016-1006-x 27785677 PMC5344946

[25] Buss SJ Krautz B Hofmann N Sander Y Rust L Giusca S. Prediction of functional recovery by cardiac magnetic resonance feature tracking imaging in first time ST-elevation myocardial infarction. Comparison to infarct size and transmurality by late gadolinium enhancement. Int J Cardiol. (2015) 183:162–70. 10.1016/j.ijcard.2015.01.022 25675901

[26] Mordi I Bezerra H Carrick D Tzemos N. The combined incremental prognostic value of LVEF, late gadolinium enhancement, and global circumferential strain assessed by CMR. JACC Cardiovasc Imaging. (2015) 8:540–9. 10.1016/j.jcmg.2015.02.005 25890580

[27] Masci PG Ganame J Francone M Desmet W Lorenzoni V Iacucci I. Relationship between location and size of myocardial infarction and their reciprocal influences on post-infarction left ventricular remodelling. Eur Heart J. (2011) 32:1640–8. 10.1093/eurheartj/ehr064 21398642

[28] Basili S Tanzilli G Raparelli V Calvieri C Pignatelli P Carnevale R. Aspirin reload before elective percutaneous coronary intervention: impact on serum thromboxane b2 and myocardial reperfusion indexes. Circ Cardiovasc Interv. (2014) 7:577–84. 10.1161/CIRCINTERVENTIONS.113.001197 25074252

[29] Adamek A Hu K Bayer B Wagner H Ertl G Bauersachs J. High dose aspirin and left ventricular remodeling after myocardial infarction: aspirin and myocardial infarction. Basic Res Cardiol. (2007) 102:334–40. 10.1007/s00395-007-0647-2 17340057

[30] Saito T Rodger IW Hu F Robinson R Huynh T Giaid A. Inhibition of COX pathway in experimental myocardial infarction. J Mol Cell Cardiol. (2004) 37:71–7. 10.1016/j.yjmcc.2004.04.002 15242737

[31] Muller DN Heissmeyer V Dechend R Hampich F Park JK Fiebeler A. Aspirin inhibits NF-kappaB and protects from angiotensin II-induced organ damage. FASEB J. (2001) 15:1822–4. 10.1096/fj.00-0843fje 11481242

[32] Podlesnikar T Pizarro G Fernandez-Jimenez R Montero-Cabezas JM Sanchez-Gonzalez J Bucciarelli-Ducci C. Five-year outcomes and prognostic value of feature-tracking cardiovascular magnetic resonance in patients receiving early prereperfusion metoprolol in acute myocardial infarction. Am J Cardiol. (2020) 133:39–47. 10.1016/j.amjcard.2020.07.037 32819681

[33] Berger JS Stebbins A Granger CB Ohman EM Armstrong PW Van de Werf F. Initial aspirin dose and outcome among ST-elevation myocardial infarction patients treated with fibrinolytic therapy. Circulation. (2008) 117:192–9. 10.1161/CIRCULATIONAHA.107.729558 18086929

[34] Mehta SR Tanguay JF Eikelboom JW Jolly SS Joyner CD Granger CB. Double-dose versus standard-dose clopidogrel and high-dose versus low-dose aspirin in individuals undergoing percutaneous coronary intervention for acute coronary syndromes (CURRENT-OASIS 7): a randomised factorial trial. Lancet. (2010) 376:1233–43. 10.1016/S0140-6736(10)61088-4 20817281

[35] Rossi R Bagnacani A Sgura F Enrique Monopoli D Coppi F Talarico M. Effect on mortality of different routes of administration and loading dose of aspirin in patients with ST-segment elevation acute myocardial infarction treated with primary angioplasty. Coron Artery Dis. (2020) 31:348–53. 10.1097/MCA.0000000000000840 31917691

[36] Zeymer U Hohlfeld T Vom Dahl J Erbel R Munzel T Zahn R. Prospective, randomised trial of the time dependent antiplatelet effects of 500 mg and 250 mg acetylsalicylic acid i.v and 300 mg p o in ACS (ACUTE). Thromb Haemost. (2017) 117:625–35. 10.1160/TH16-08-0650 28102427

[37] Park Y Tantry US Koh JS Ahn JH Kang MG Kim KH. Novel role of platelet reactivity in adverse left ventricular remodelling after ST-segment elevation myocardial infarction: the REMODELING Trial. Thromb Haemost. (2017) 117:911–22. 10.1160/TH16-10-0744 28150852

[38] Dillinger JG Saeed A Spagnoli V Sollier CB Sideris G Silberman SM. High platelet reactivity on aspirin in patients with acute ST elevation myocardial infarction. Thromb Res. (2016) 144:56–61. 10.1016/j.thromres.2016.05.002 27289074

[39] Niccoli G Giubilato S Russo E Spaziani C Leo A Porto I. Plasma levels of thromboxane A2 on admission are associated with no-reflow after primary percutaneous coronary intervention. Eur Heart J. (2008) 29:1843–50. 10.1093/eurheartj/ehn325 18617477

