# Supplementary material for: Protective Value of Aspirin Loading Dose on Left Ventricular Remodeling After ST-Elevation Myocardial Infarction
Source: Front Cardiovasc Med. 2022 Mar 16;9:786509. doi: 10.3389/fcvm.2022.786509 (PMC8965885; doi:10.3389/fcvm.2022.786509)
Supplement: Supplementary file 1 [file Table_1.DOCX]

**Table 6. Intra-observer and inter-observer variability for measurements of GRS, GCS and GLS**

| **GCS** | Intraobserver   ICC  0.931, p < 0.001 |
| --- | --- |
|  | Interobserver  ICC  0.922, p < 0.001 |
| **GLS** | Intraobserver   ICC 0.928, p < 0.001 |
|  | Interobserver   ICC 0.927, p < 0.001 |
| **GRS** | Intraobserver   ICC  0.943, p < 0.001 |
|  | Interobserver   ICC  0.925, p < 0.001 |
